# Stiff‐person syndrome revealing an occult gray zone lymphoma: A diagnostic challenge

**DOI:** 10.1002/ccr3.8328

**Published:** 2023-12-14

**Authors:** Mohammad Sadegh Fakhari, Leila Poorsaadat, Hooman Mohammad Talebi, Farid Kosari

**Affiliations:** ^1^ Student Research Committee Arak University of Medical Sciences Arak Iran; ^2^ Department of Neurology, School of Medicine Arak University of Medical Sciences Arak Iran; ^3^ Nursing and Midwifery Faculty Isfahan University of medical Sciences Isfahan Iran; ^4^ Department of Pathology, School of Medicine Tehran University of Medical Sciences Tehran Iran

**Keywords:** gray zone lymphoma, paraneoplastic syndrome, stiff‐person syndrome

## Abstract

**Key Clinical Message:**

Stiff‐Person Syndrome (SPS) can be associated with various malignancies, including lymphomas. Therefore, clinicians should always remain vigilant for the presence of an underlying malignancy, especially in older patients presenting with SPS.

**Abstract:**

Stiff‐person syndrome (SPS) is a rare neurological disorder characterized by painful muscle spasms. It can occur as a paraneoplastic syndrome associated with various malignancies. We present a case of an older male adult with a history of recurrent fever episodes and elevated inflammatory markers for 1 year who subsequently developed neurological symptoms. The presence of positive amphiphysin antibodies led to the diagnosis of SPS, which prompted further investigations revealing an underlying Gray Zone lymphoma (GZL). This case highlights the challenges in diagnosing lymphoma and emphasizes the importance of considering SPS as a paraneoplastic syndrome in guiding toward the final diagnosis. The diagnostic challenge in our case is summarized in Chart 1.

## Graphical abstract

Summary of the diagnostic dilemma.

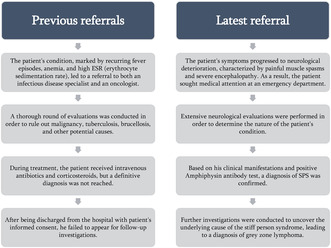

## 1 INTRODUCTION

Stiff‐person syndrome (SPS) is a rare condition that falls under the category of neuroimmunological disorders.^1^ The diagnosis of this disease can be challenging due to the various clinical and laboratory factors involved. Autoantibodies against glutamic acid decarboxylase (GAD), which is the GABA synthesizing enzyme are found in up to 80% of SPS patients.^2^ Common characteristics of SPS include severe stiffness in the neck, shoulder, and lower extremities, as well as episodic spasms in muscles that are worsened by stress and other triggers. Research indicates that autoimmune conditions and paraneoplastic disorders are the most prevalent causes of SPS. Considering autoimmune disease and paraneoplastic syndromes as the main causes of SPS, this reveals the importance of further investigation to determine the underlying etiology.^3^, ^4^

In this article, we report a case of an older adult who was admitted to the emergency department complaining of painful muscle spasms in his lower limbs. The patient had a history of recurrent, episodic fevers. Routine clinical and laboratory tests were performed, but no infectious or oncologic results were found. Subsequently, further pathologic tests were conducted to investigate the causes of SPS, which led to evidence of gray zone lymphoma (GZL).

## 2 CASE PRESENTATION

A 66‐year‐old educated male is presented to the emergency department with painful muscle spasms in his lower limbs that have been occurring for a few days. He initially experienced difficulty walking and episodic limb pain, and he noticed a slowness in his movements a month ago. Additionally, he complained of a lack of concentration and memory loss over the past week. He had no history of trauma, loss of consciousness, headache, or vomiting. He had no history of surgery, allergies, addiction, or smoking.

However, he had a history of anemia, which was initially diagnosed as iron deficiency anemia. His evaluations did not reveal any evidence of gastrointestinal malignancies, and he has been treated with daily ferrous sulfate tablets. Furthermore, over the past year, he experienced recurrent episodes of weakness and fever with an almost 12‐kg weight loss, leading to referrals to an infectious disease specialist. During these periods, he underwent evaluations for oncologic and infectious diseases, but no specific final diagnosis was reached. As a result, he was hospitalized multiple times and received treatment with intravenous antibiotics and corticosteroids. His medical files include investigations from his prior referral to hospital. These investigations are summarized in the next chapter.

Upon admission, his general condition was poor; he was confused and did not demonstrate signs of orientation to time and person. His vital signs were within the normal range. In his general examinations, there was no indication of icterus, cyanosis, lymphadenopathy, edema, or clubbing. However, severe conjunctival pallor is observed. On neurologic examinations, his coordination and sensation were normal. Cranial nerve examinations revealed normal results, except for signs of vertical gaze impairment. Higher mental function examinations indicated severe encephalopathy, with a Mini‐Mental State Examination (MMSE) score of 18. Motor examinations showed normal muscle bulk and power (force = 5/5) in both upper and lower limbs, while bilaterally increased tone was observed in both the upper and lower limbs. His gate examinations revealed severe bradykinesia with a spastic gate. No other abnormal movement was observed. His plantar reflex examination showed an upward response. Other neurological examinations were normal.

## 3 INVESTIGATIONS

As previously mentioned, the patient has undergone multiple referrals to oncologists and infectious disease specialists due to recurrent episodes of fever over the past year. These evaluations were conducted prior to referral to the neurologist. The patient's medical records include investigations from 3 months ago, when he was admitted to the hospital due to fever. These investigations are summarized in Table 1.

**TABLE 1:** Previous investigations.

	Unit	Result	Normal range
Serum analysis			
White blood cell count	Per mm^3^ ×10^3^	6.050	4–10
Hemoglobin	mg/dL	9.8	13.5–18
Hematocrit	%	28.6	42–52
Mean corpuscular volume	FL	82.7	77–97
Platelet count	Per mm^3^ ×10^3^	240	140–440
Urea	mg/dL	47.9	13–43
Creatinine	mg/dL	1.11	0.8–1.3
Sodium	mg/dL	138	136–145
Potassium	mg/dL	3.6	3.5–5.1
Calcium	mg/dL	9.4	8.5–11
Phosphorus	mg/dL	3.6	2.5–4.5
Magnesium	mg/dL	2.1	1.7–2.4
Ferritin	ng/mL	>400	20–250
Iron	μg/dL	21	30–200
Lead	μg/dL	5.0	<20
Total iron BINDING capacity	Ug/dL	225	250–450
1‐Hour ESR	mm	100	<20
C‐reactive protein	–	2+	Negative
Aspartate aminotransferase	IU/L	52	4–37
Alanine transaminase	U/L	38	4–37
Alkaline phosphatase	IU/L	350	80–306
Total serum bilirubin	mg/dL	1.45	0.3–1.5
Thyroid stimulating hormone	μIU/ml	1.81	0.39–6.16
Thyroxin (total T4 hormone)	μg/dL	7.12	5–10.07
Blood culture	–	No growth after 72 hours	
Urine analysis			
Protein	–	1+	Negative
Hemoglobin	–	2+	Negative
White blood cell	Count	2–3	<4
Red blood cells	Count	14–16	<4
Urine Culture	–	No growth after 48 hours	
Stool examination			
White blood cell	–	Not seen	
Red blood cells	–	Not seen	
Occult blood	–	Not seen	
Fat droplets	–	Not seen	

Serum protein electrophoresis showed polyclonal hypergammaglubolinemia and an increase in Alpha 1 (0.6 g/dL), Alpha 2 (1.1 g/dL), and Beta 2 (0.6 g/dL) proteins. Urine protein electrophoresis showed tubular albuminuria (12.8 mg/dL).

The abdomino‐pelvic ultrasound study revealed normal results. The chest computed tomography (CT) scan showed mild consolidations in the inferior‐posterior lobes of both lungs but was otherwise unremarkable. Subsequently, an endoscopy was performed, which indicated mild chronic erosive gastritis without intestinal metaplasia, peptic ulcer, or *Helicobacter pylori* infection. The colonoscopy study yielded normal findings.

The investigations concluded with a bone marrow aspiration and biopsy, which revealed a normocellular marrow with trilineage hematopoiesis and progressive maturation. The presence of plasma cells was noted at a level of 5%–7%.

Following these investigations, the patient's symptoms improved with the administration of intravenous antibiotics and corticosteroids, leading to his discharge from the hospital with the patient's informed consent. Unfortunately, the patient did not show up for further follow‐up. His symptoms eventually progressed to neurologic deterioration, prompting him to seek medical attention at the emergency department. He presented with painful spasms and signs of severe encephalopathy.

Upon referral to the neurologist in the latest admission, a thorough neurological evaluation was done for the patient. His general laboratory findings were consistent with previously mentioned results, except for a lower hemoglobin level (7.6 mg/dL). Further investigations included a lumbar puncture, Immunologic tests, and paraneoplastic panel. These are summarized in Table 2.

**TABLE 2:** Further investigations.

	unit	Result	Normal range
CSF analysis			
Lactate dehydrogenase	U/L	45	<70
Glucose	mg/dL	58	45–100
White blood cell	Per mm^3^	0	<5
Red blood cell	Per mm^3^	0	0
Protein	mg/dL	45	14–45
CSF culture	–	No growth after 72 hours	
Polymerase chain reaction for	–	negative	Negative
HSV, CMV, EBV, and VZV			
Immunology			
Adenosine deaminase	U/L	7	0–15
Rheumatic factor	–	Positive	negative
Antinuclear antibody	IU/mL	13	8
Peripheral anti‐neutrophil cytoplasmic Antibodies (P‐ANCA)	IU/mL	<3	<12.0
Cytoplasmic anti‐neutrophil cytoplasmic Antibodies (C‐ANCA)	IU/mL	23.8	<10
Anti‐Ds DNA	Ratio	0.36	<1.0
Prostate specific antigen	ng/ml	1.12	0–4
Wright agglutination test	Titer	Negative	Negative
2‐mercaptoethanol test	Titer	Negative	Negative
Coombs wright	Titer	Negative	Negative
β2 microglobulin	mg/L	5	1.0–3.0
Brucella igg	Index	0.33	<9.0
Tuberculin skin test	mm	1	<5
HTLV 1 & 2 serology	–	0.17	<0.9
Antigens Of paraneoplastic neurologic syndromes			
Amphiphysin	–	64 (3+)	0 (Negative)
Glutamic acid decarboxylase antibody 65 (GAD 65)	–	Negative	Negative

Brain magnetic resonance imaging (MRI) did not reveal any abnormalities. However, a whole spine MRI study was conducted, which indicated moderate canal stenosis with pressure effect at the L5‐S1 level. This finding could potentially explain the upward plantar reflex observed in the patient, although a more complex diagnosis was anticipated in this case.

Electroencephalography (EEG) results demonstrated a transient slowing pattern and some epileptic discharges.

The paraneoplastic panel study revealed an increase in amphiphysin antigen, which supported a diagnosis of SPS. To further investigate the underlying cause of SPS, an autoimmune encephalitis panel was conducted, including testing for GABAB receptor and anti‐glutamate receptor antibodies. However, these tests yielded negative results.

Given the negative autoimmune encephalitis panel findings, attention was directed toward paraneoplastic etiologies of SPS, especially considering the patient's significant weight loss. A breast ultrasound study was performed, which showed normal results. Additionally, a whole‐body bone scan indicated areas of focal uptake at the left fifth and tenth ribs. These findings were considered nonspecific and could potentially be attributed to either fractures or metastasis.

**FIGURE 1:**
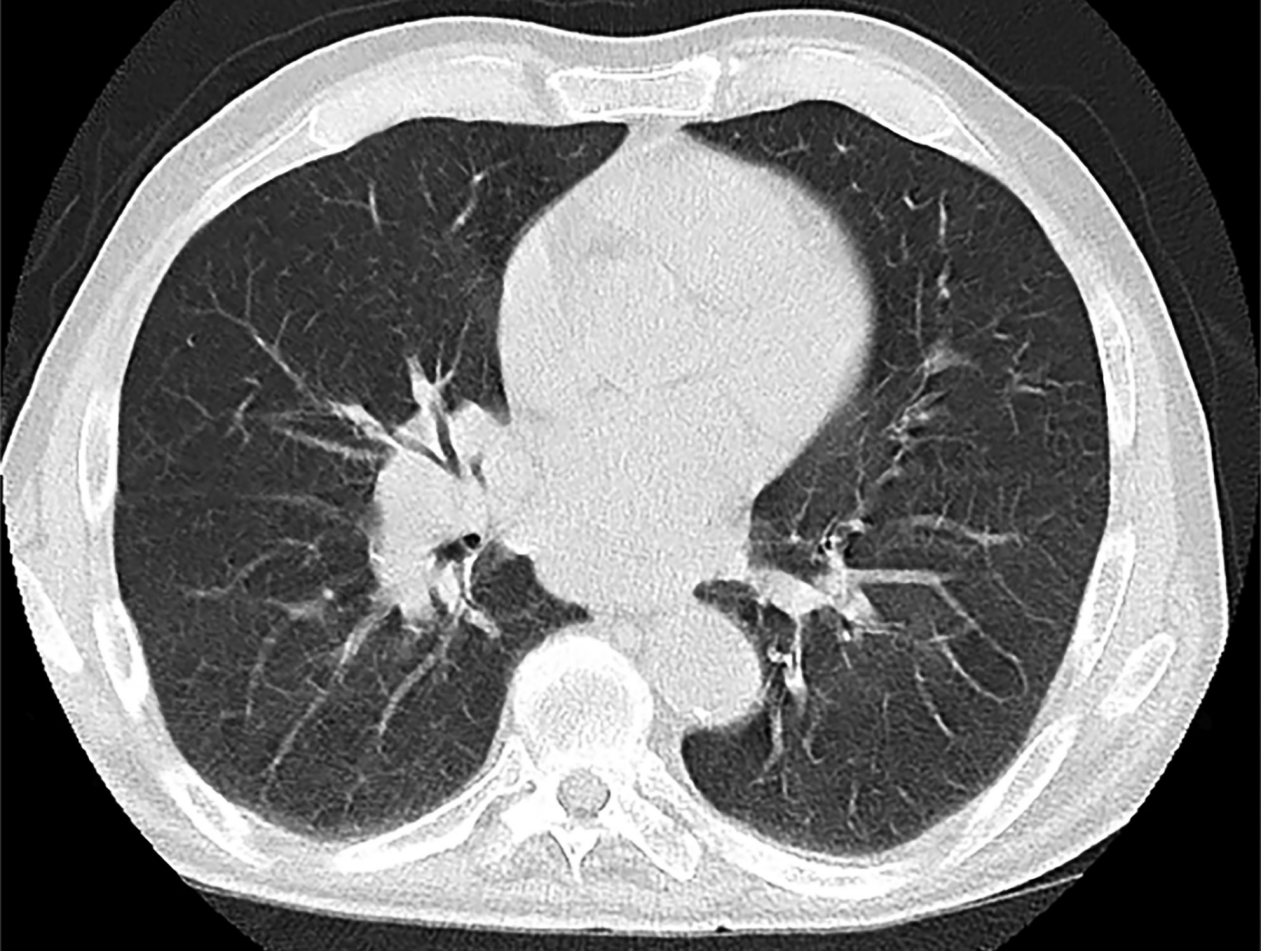
High‐resolution CT (HRCT) of the chest showing hilar lymphadenopathy.

Despite the previous chest CT scan, a second high‐resolution CT (HRCT) of the chest was conducted. The HRCT report revealed a soft tissue density measuring 48 × 21 mm in the subcarinal space, along with several lymphadenopathies with a maximum short‐axis diameter of 14 mm, which is depicted in Figure 1. Additionally, a thin rim of pleural effusion was observed on the right side, accompanied by mild atelectasis at the base of both lungs.

To further investigate the possibility of malignancy, bronchoalveolar lavage (BAL) cytology was performed. However, the results of the BAL cytology did not indicate the presence of malignant cells.

A needle biopsy of the mediastinal lymph node was performed and sent for microscopic examination. However, the specimen was deemed inadequate to definitively rule in or rule out lymphoproliferative disorders. The report indicated a polytypic lymphoid cell population with some anthracotic pigments present. Given the high clinical suspicion, the specimen was sent to another laboratory for a second review, along with immunohistochemistry (IHC) studies.

**FIGURE 2:**
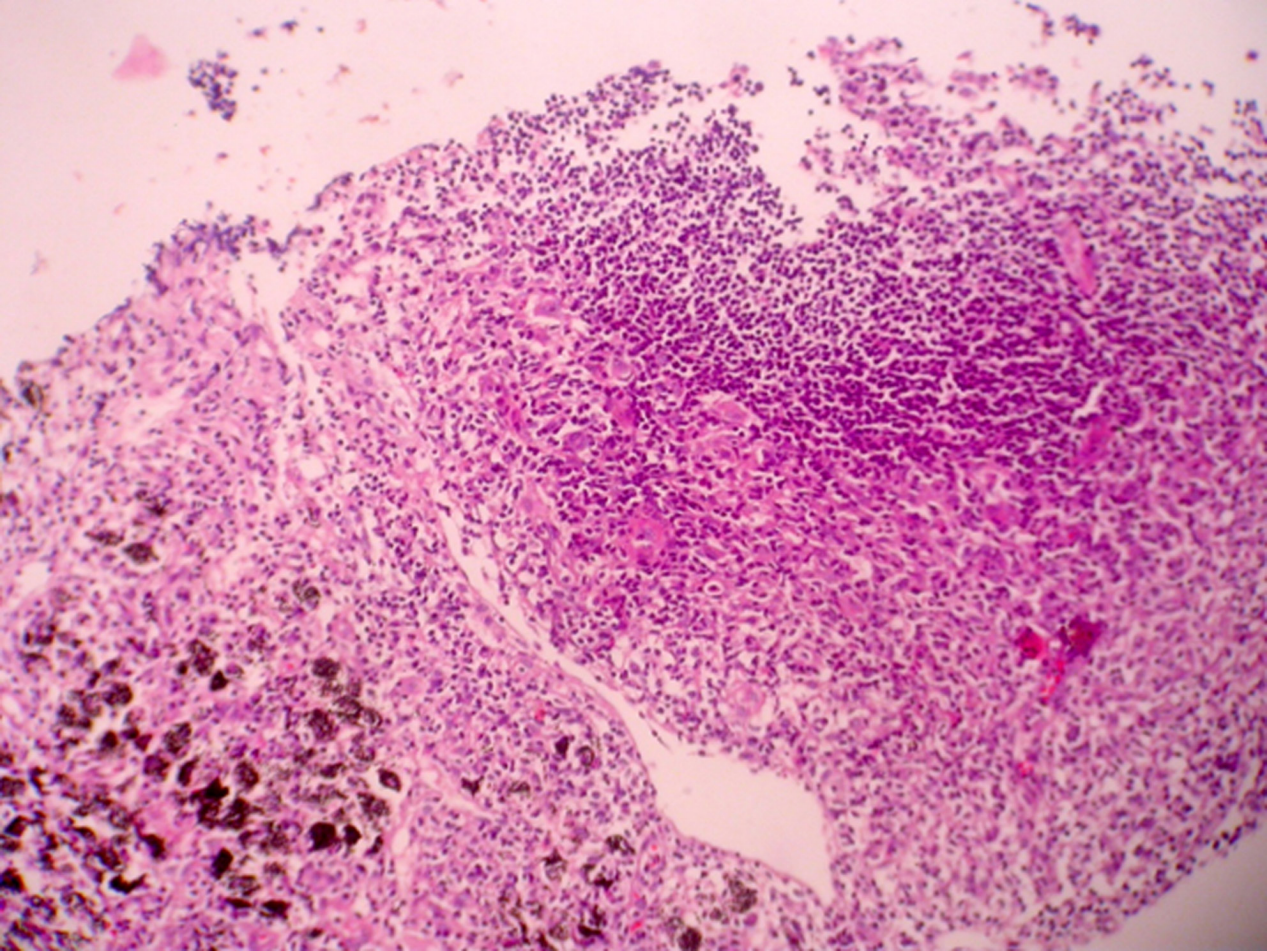
Low power view of the core needle biopsy showing small fragment of lymphoid tissue with anthracosis, containing polymphic lymphoid cells. (H&E × 100).

**FIGURE 3:**
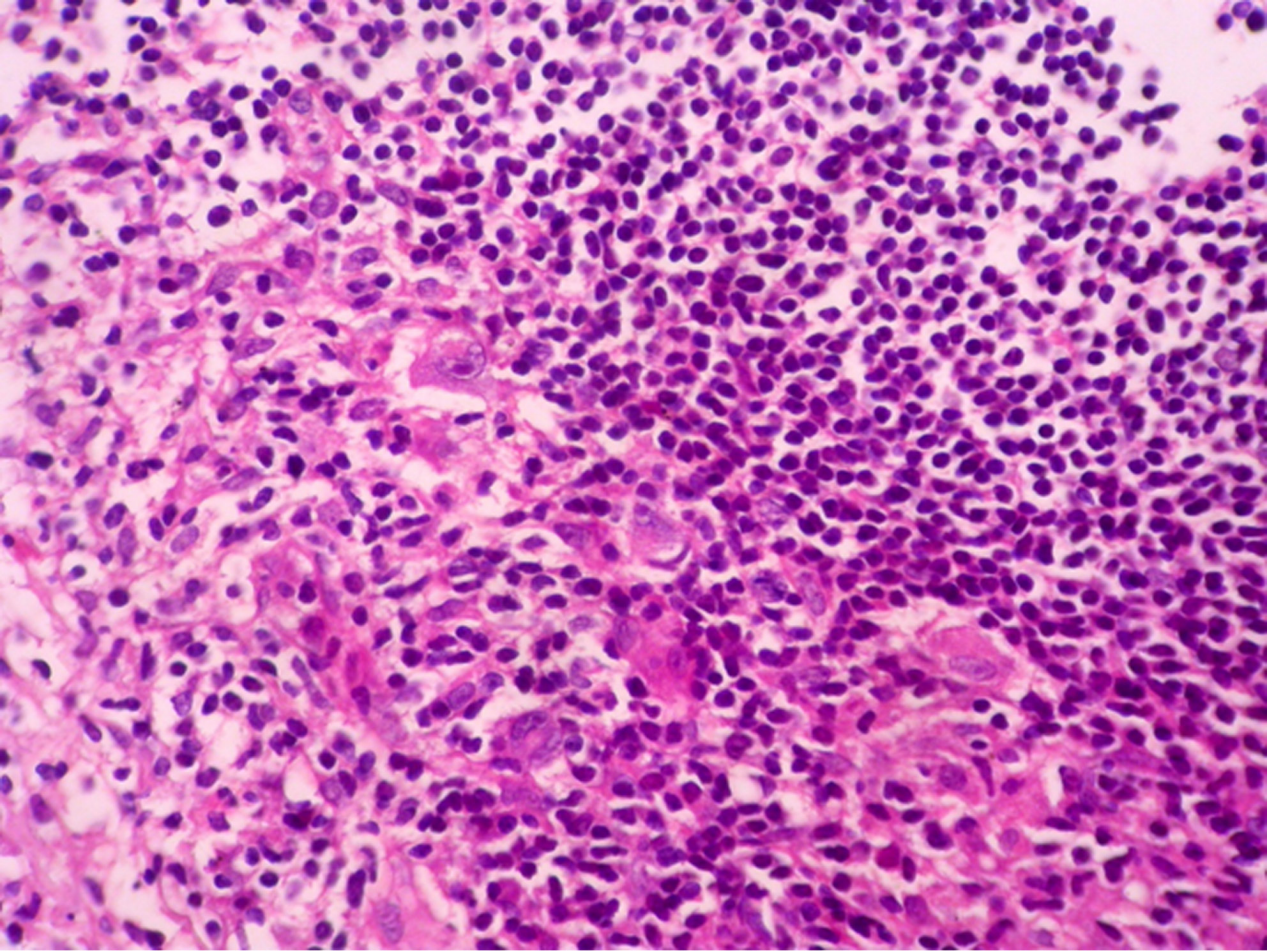
High power view of the lymphoid tissue showing many large lymphoid cells resembling Hodgkin cells and Red Sternberg cells. (H&E × 200).

**FIGURE 4:**
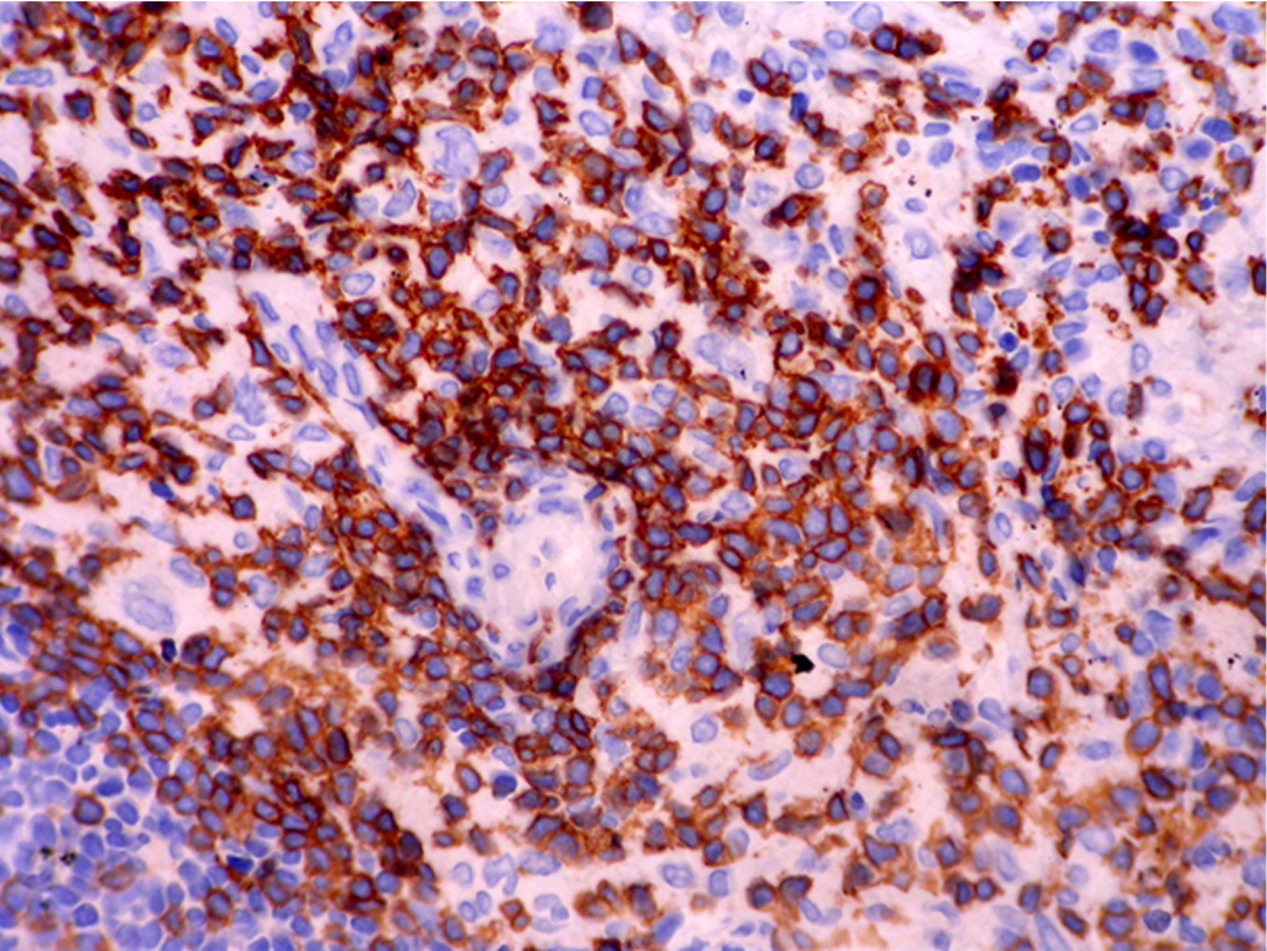
Immunohistochemical staining for CD3, showing positive staining in background small lymphocytes and negative staining of large, atypical cells.

**FIGURE 5:**
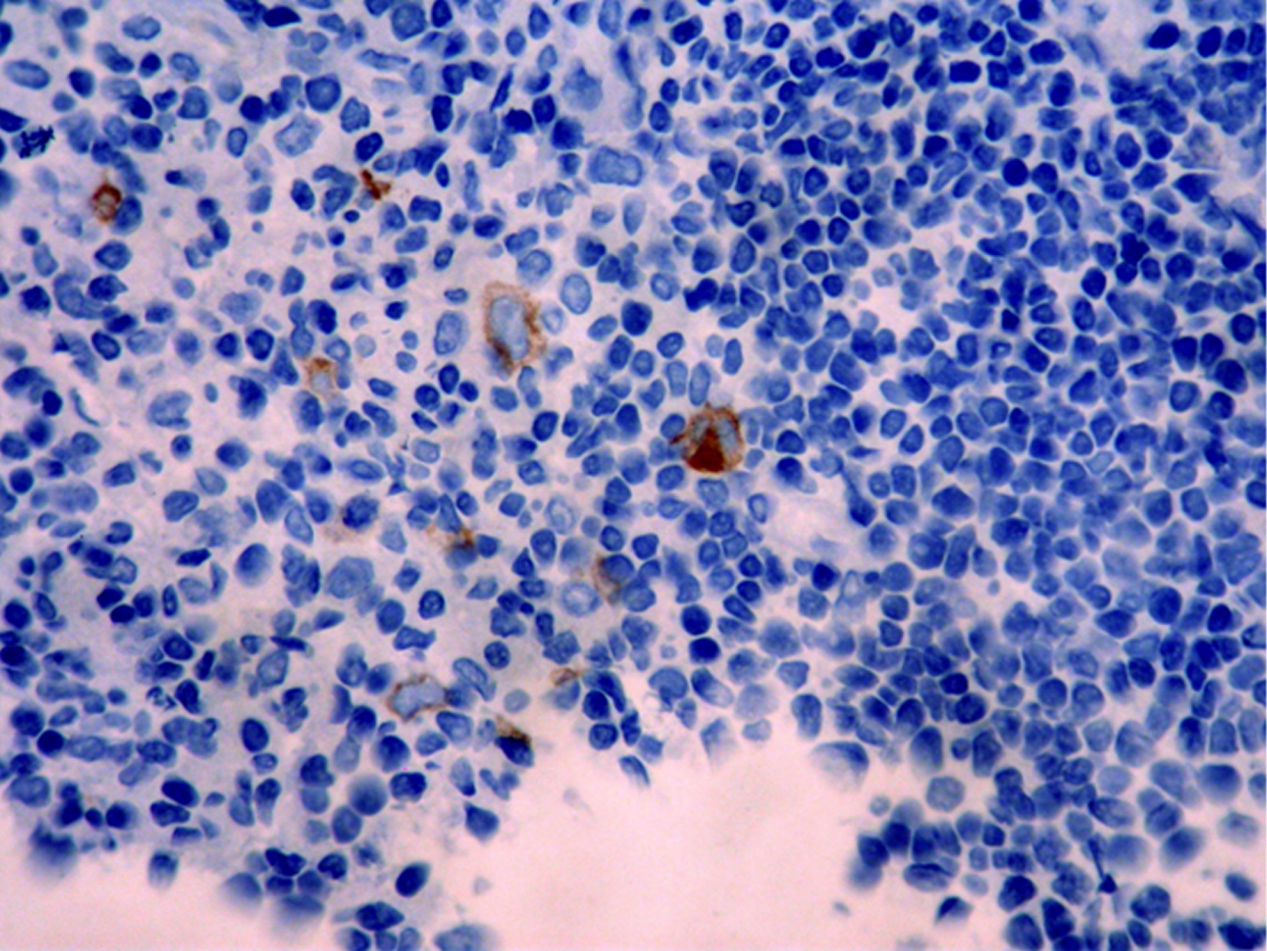
Immunohistochemical staining for CD30, showing positive staining of large lymphoid cells.

**FIGURE 6:**
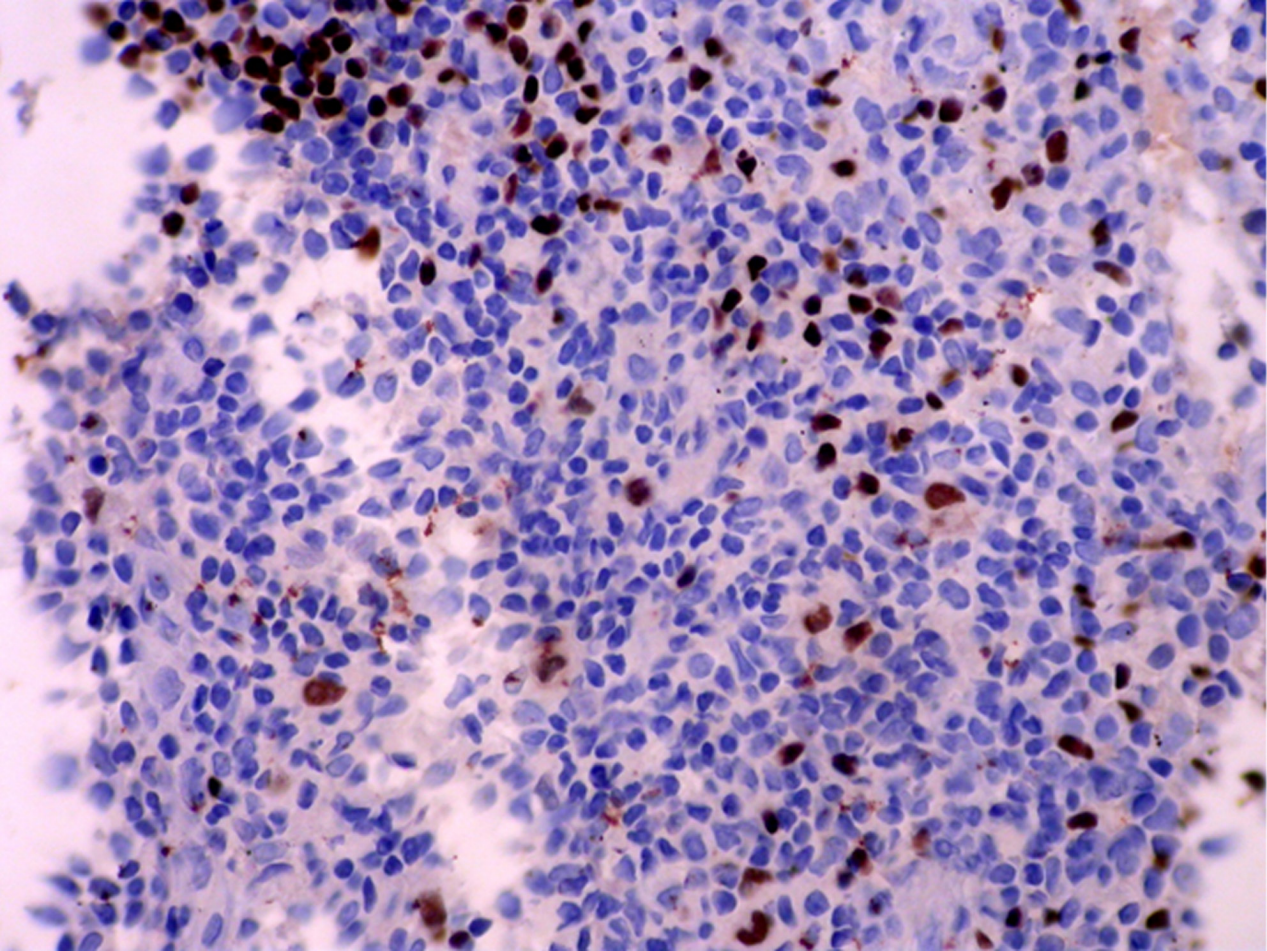
Immunohistochemical staining for PAX5, showing positive staining in large, atypical cells.

**FIGURE 7:**
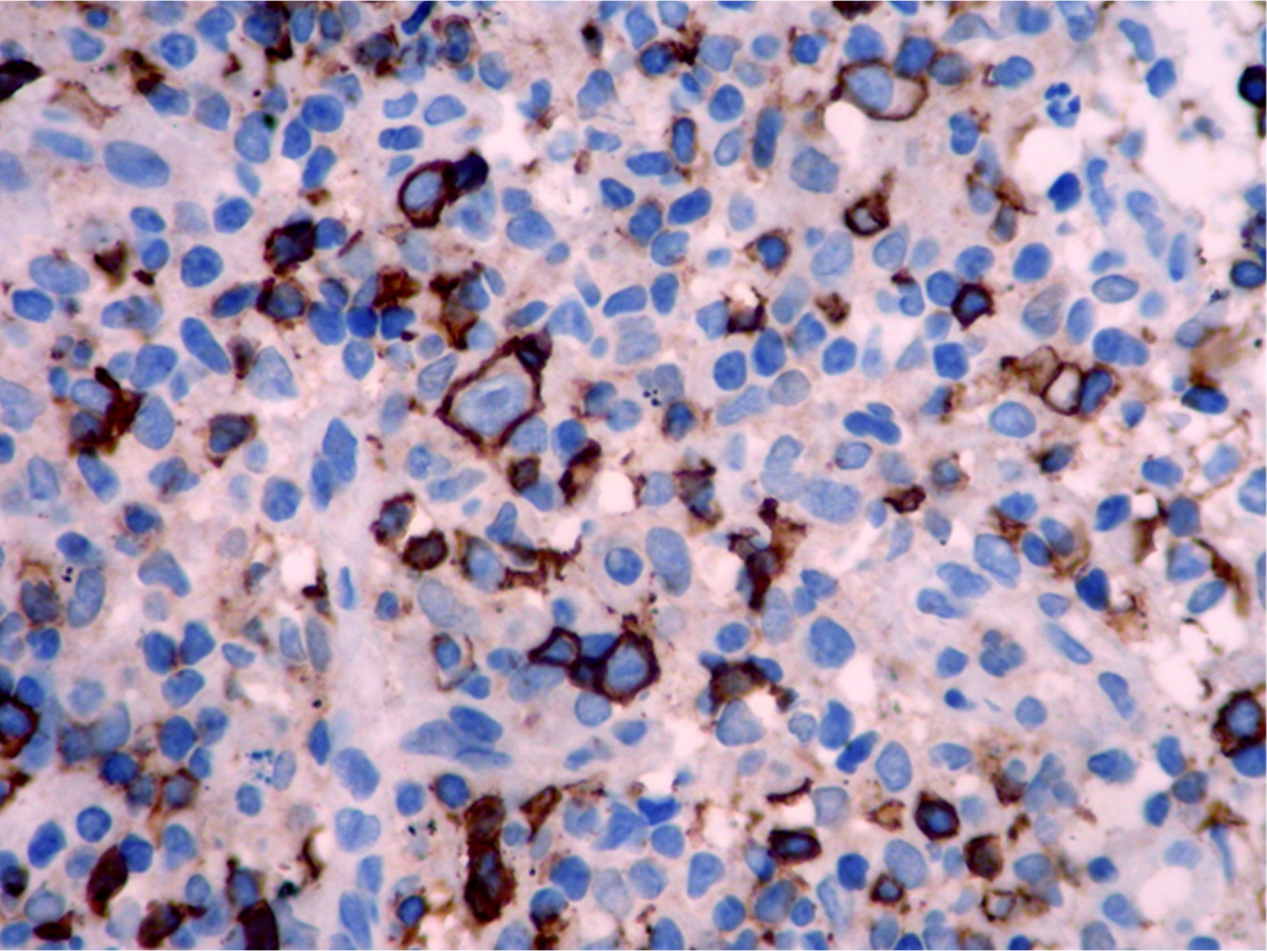
Immunohistochemical staining for CD20 which is positive in some background small lymphocytes and large, atypical cells.

Histopathologic features of core needle biopsy show tiny fragments of lymphoid tissue with anthracosis, containing polymorphic lymphoid cells composed of small lymphocytes with few large atypical lymphoid cells resembling Hodgkin cell and Red‐Sternberg cells in between (Figures 2 and 3). On IHC staining, the background small lymphocytes are positive for CD3 (Figure 4), and the mentioned large atypical cells are positive for CD30 (Figure 5), PAX5 (Figure 6), and CD20 (Figure 7). They show negative staining for CD15. Based on the morphological and IHC findings, the diagnosis of GZL was favored.

## 4 DIFFERENTIAL DIAGNOSIS

Due to the warning signs in this patient (an older adult with fever and weight loss), the possibility of a malignancy was high. As previously mentioned, previous investigations were done to rule out possible malignancies including gastrointestinal malignancies, breast cancer, lung cancer, leukemia, lymphoma, and multiple myeloma. However, these investigations were all negative.

Upon his referral to neurologist with painful muscle spasm, severe encephalopathy, and fever, several potential diagnoses were considered, including meningoencephalitis, neuroleptic malignant syndrome (NMS), parkinsonian disorders, progressive supranuclear palsy (PSP), and paraneoplastic syndromes.

However, among these diagnoses, paraneoplastic syndromes were more possible due to the phenotype of SPS, high clinical suspicion for a malignancy, and positive amphiphysin antigen.

## 5 TREATMENT

As EEG results demonstrated epileptic discharges, levetiracetam at a dosage of 500 mg twice a day was started. Furthermore, given the patient's painful spasms, treatment was initiated with oral Baclofen at a dosage of 5 mg three times a day, along with Clobazam at a daily dose of 10 mg.

The strong clinical suspicion of paraneoplastic syndrome was supported by the previously introduced paraneoplastic syndrome care score.^5^ The patient exhibited the SPS phenotype and tested positive for amphiphysin antibody without a diagnosed cancer after less than 2 years of follow‐up, achieving a score of 6 out of 8 points. This indicates a probable diagnosis of paraneoplastic syndrome. Consequently, treatment with intravenous immunoglobulin (IVIG) therapy was initiated at a dosage of 0.4 g/kg per day for 5 days following the detection of amphiphysin antibody results.^6^, ^7^ However, since there was also a high clinical suspicion of malignancies, corticosteroid treatment was not initiated. These treatments were accompanied by a series of neurological evaluations aimed at reaching a definitive diagnosis.

## 6 OUTCOME

Signs and symptoms of SPS subsided following treatment with Baclofen and Clobazam. The patient did not experience seizure, despite having epileptic discharges in the EEG results. Furthermore, the patient's cognition improved after treatment with IVIG, and the MMSE score increased to 25.

However, due to the lengthy process involved in obtaining a conclusive diagnosis and the rapid progression of the disease, the patient's condition deteriorated further. Tragically, the patient succumbed to the illness before treatment for the malignancy could be commenced.

## 7 DISCUSSION

Stiff‐person syndrome is an uncommon neurological autoimmune syndrome, with an incidence rate of one case per million.^1^ This syndrome can be categorized into three subgroups based on its etiology: autoimmune, paraneoplastic, and cryptogenic. Paraneoplastic SPS presents with a broad range of clinical manifestations, including weakness, seizures, gait abnormalities, diplopia, and myoclonus.^8^ Other paraneoplastic syndromes that share similarities with SPS include Opsoclonus myoclonus syndrome and progressive encephalomyelitis with rigidity and myoclonus.^8^

The diagnostic criteria for SPS, which have gained widespread acceptance, were revised by Dalakas et al. in 2009. These criteria encompass both clinical and paraclinical indicators.^9^ In our case, we observed an older male adult presenting with painful muscle spasms, positive serology for anti‐amphiphysin autoantibodies, a positive clinical response to benzodiazepines, and the absence of any other neurological diagnosis that could account for his signs and symptoms. As a result, this case meets four out of the six criteria established by Dalakas et al.^9^

Based on research findings, it has been determined that approximately 5% of SPS cases are paraneoplastic, and these cases are often linked to breast cancer or small‐cell lung cancer.^10^ Nevertheless, there have been documented cases of SPS associated with Hodgkin lymphoma.^8^ To the best of our knowledge, there have been no reported instances of coexisting SPS and GZL.

Notably, the majority of case reports regarding paraneoplastic SPS focus on patients who develop this syndrome following their cancer diagnosis. In contrast, our case was diagnosed with SPS before the discovery of cancer, underscoring the significance of SPS as a potential early indicator of the underlying cause. However, in our case, the presenting symptoms primarily raised clinical suspicion of malignancy, as the patient was an older adult experiencing fever and weight loss. Furthermore, in this case, despite multiple consultations with infectious disease specialists and oncologists, he only received intravenous antibiotics and corticosteroid treatment without a definitive diagnostic plan to identify the underlying etiology of the symptoms.

The coexistence of SPS and GZL in this case is also notable, given the rarity and diagnostic challenges associated with both conditions. GZL, in particular, is a rare form of lymphoma that presents a diagnostic and clinical dilemma for pathologists due to its complex morphological and phenotypic characteristics. Oncologists also face challenges in managing GZL due to its aggressive nature and the lack of well‐established treatment guidelines.^11^

In conclusion, this case emphasizes the importance of a comprehensive evaluation of patients presenting with SPS to uncover any underlying conditions, even when the symptoms of SPS overshadow other clinical manifestations. This underscores the necessity of a multidisciplinary approach to arrive at a definitive diagnosis.

## AUTHOR CONTRIBUTIONS

**Mohammad Sadegh Fakhari:** Conceptualization; data curation; formal analysis; investigation; methodology; resources; writing – original draft; writing – review and editing. **Leila Poorsaadat:** Conceptualization; data curation; formal analysis; investigation; methodology; project administration; resources; software; supervision; validation; visualization; writing – review and editing. **Hooman Mohammad talebi:** Conceptualization; data curation; writing – original draft; writing – review and editing. **Farid Kosari:** Conceptualization; data curation; investigation; methodology; resources; supervision; validation; visualization; writing – review and editing.

## FUNDING INFORMATION

This article was no funded by any individual or organization.

## CONFLICT OF INTEREST STATEMENT

All the authors declared no conflict of interest.

## ETHICS STATEMENT

A written informed consent was obtained from the next of kin. Authors confirm that all methods were performed in accordance with institutional ethical standards and the Declaration of Helsinki.

## CONSENT

Written informed consent was obtained from the patient to publish this report in accordance with the journal's patient consent policy.

## Data Availability

All data are available from the corresponding author on reasonable request.
